# The structure of His-tagged *Geobacillus stearothermophilus* purine nucleoside phosphorylase reveals a ‘spanner in the works’

**DOI:** 10.1107/S2053230X22011025

**Published:** 2022-11-28

**Authors:** Fiona M. Given, Fuchsia Moran, Ashleigh S. Johns, James A. Titterington, Timothy M. Allison, Deborah L. Crittenden, Jodie M. Johnston

**Affiliations:** a School of Physical and Chemical Sciences, Biomolecular Interaction Centre, University of Canterbury, New Zealand; Baylor College of Medicine, Houston, USA

**Keywords:** purine nucleoside phosphorylase, *Geobacillus stearothermophilus*, His tags, affinity tags

## Abstract

The 1.72 Å resolution structure of purine nucleoside phosphorylase from *Geobacillus stearothermophilus*, a thermostable protein of potential interest for the biocatalytic synthesis of antiviral nucleoside compounds, is reported. The structure of the N-terminally His-tagged enzyme is a hexamer, as is typical of bacterial homologues, with a trimer-of-dimers arrangement. Unexpectedly, several residues of the recombinant tobacco etch virus protease (rTEV) cleavage site from the N-terminal tag are located in the active site of the neighbouring subunit in the dimer. Key to this interaction is a tyrosine residue, which sits where the nucleoside ring of the substrate would normally be located. Tag binding appears to be driven by a combination of enthalpic, entropic and proximity effects, which convey a particularly high affinity in the crystallized form. Attempts to cleave the tag in solution yielded only a small fraction of untagged protein, suggesting that the enzyme predominantly exists in the tag-bound form in solution, preventing rTEV from accessing the cleavage site. However, the tagged protein retained some activity in solution, suggesting that the tag does not completely block the active site, but may act as a competitive inhibitor. This serves as a warning that it is prudent to establish how affinity tags may affect protein structure and function, especially for industrial biocatalytic applications that rely on the efficiency and convenience of one-pot purifications and in cases where tag removal is difficult.

## Abstract 2

The structure of *Geobacillus stearothermophilus* purine nucleoside phosphorylase, an enzyme of biocatalytic interest, is reported and was found to include the presence of an N-terminal tag in the active site of each subunit that belongs to the other subunit in each dimer. This serves as a warning that while tags enable simple and economic purification for proteins of industrial interest, care needs to be taken to ensure that they do not have unwanted effects on protein structure and function.

## 1. Introduction

**Figure 1:**
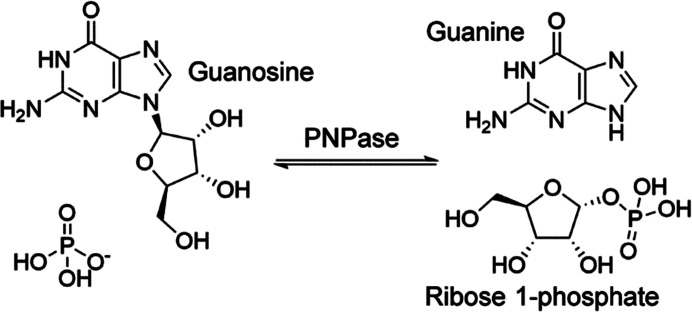
The enzymatic reaction performed by PNPase. The purine base and nucleoside can be modified to create different base/nucleoside pairs.

Purine nucleoside phosphorylase (PNPase) is an enzyme in the purine salvage pathway that is present in some organisms. This enzyme performs the reversible phosphorolysis of nucleosides to form ribose 1-phosphate and a purine base (Fig. 1 ▸; Bzowska *et al.*, 2000 ▸). There are two major structural forms of PNPase: a trimeric form primarily found in eukary­otes and a hexameric ‘trimer-of-dimers’ form primarily found in bacteria (Štefanić *et al.*, 2017 ▸). The trimeric and hexameric forms of PNPase have similar subunit structures, namely an α/β fold (Mao *et al.*, 1997 ▸), and differ primarily in substrate specificity, with most studied trimeric PNPases restricted to 6-oxopurine ribonucleosides, while hexameric PNPases can use both 6-oxopurine ribonucleosides and 6-aminopurine ribonucleosides (Bennett *et al.*, 2003 ▸; Ducati *et al.*, 2009 ▸). Whilst the hexameric form is primarily found in bacteria, many bacteria have both forms encoded in their genome, including *Geobacillus stearothermophilus*, which is a Gram-positive thermophile bacterium and a common cause of spoilage in food products (Hamamoto *et al.*, 1997 ▸).

PNPases are potential antimicrobial drug targets (Pant *et al.*, 2021 ▸; Madrid *et al.*, 2008 ▸) and industrial catalysts for the production of antiviral nucleoside compounds (Nannemann *et al.*, 2010 ▸; Xie *et al.*, 2011 ▸). The substrate promiscuity of PNPases is relevant for the latter application, as the wider the range of purine bases that a PNPase can accommodate, the broader the scope of antiviral nucleoside candidates that it may be used to produce. As many of the potential purine bases have limited solubility at ambient temperature (Zhu *et al.*, 2013 ▸), PNPases from thermophilic organisms, such as *G. stearothermophilus*, could be used at higher temperatures with consequently improved substrate solubility and thus allow the production of a wider range of these compounds. As part of our exploration of the biocatalytic potential of thermo­stable PNPases, we solved the structure of the N-terminally His-tagged PNPase from *G. stearothermophilus* (His-*Gse*PNPase).

## 2. Materials and methods

### 2.1. Macromolecule production

The His-*Gse*PNPase gene from *G. stearothermophilus* (KEGG GT50_19015[TA1]) was purchased as a synthetic gene from GenScript. The gene was amplified and inserted into a pET-30a expression vector by In-Fusion cloning (Park *et al.*, 2015 ▸) using NcoI and XhoI restriction sites. The resulting plasmid encodes an N-terminal 6×His tag, a thrombin cleav­age site, an S-tag followed by a tobacco etch virus protease (TEV) recognition sequence, and the opening reading frame of interest.

The purified plasmid was transformed into chemically competent *Escherichia coli* BL21 (DE3) cells for protein expression. The cultures were grown at 37°C in lysogeny broth medium with 100 µ*M* kanamycin until the optical density was in the range 0.4–0.6; protein expression was then induced using isopropyl β-d-1-thiogalactopyranoside at a final concentration of 100 µ*M* and the cells were grown overnight at 18°C. The cells were harvested by centrifugation at 12 000*g* for 15 min and the resultant cell pellets were stored at −80°C until use.

The cell pellet was resuspended in lysis buffer (50 m*M* HEPES pH 7.6, 100 m*M* NaCl, 20 m*M* imidazole) and lysed by sonication. Following centrifugation, the supernatant was filtered through a 0.22 µm filter and then purified by immobilized metal-affinity chromatography (IMAC) on a HisTrap FF column (Cytiva). The protein was eluted from the column using a gradient of elution buffer (50 m*M* HEPES pH 7.6, 100 m*M* NaCl, 500 m*M* imidazole). Size-exclusion chromatography on a Superdex 10/300 column run in storage buffer (50 m*M* HEPES pH 7.6, 100 m*M* NaCl) was then used to further purify the protein prior to crystallography. Purified protein was stored at −80°C until use. Protein concentration was determined from the UV absorbance in a NanoDrop spectrophotometer (ThermoFisher) using a theoretical molar extinction coefficient of 27 515 *M* ^−1^ cm^−1^ as determined by *ProtParam* (Gasteiger *et al.*, 2005 ▸).

When cleavage by recombinant TEV (rTEV) was attempted, 1 m*M* tris(2-carboxyethyl)phosphine (TCEP) was added. Purified rTEV was added at a ratio of 1:10 rTEV:protein. The sample was left overnight at 4°C or incubated at 37°C for 4 h and then incubated at 4°C overnight before a second IMAC purification was performed. Macromolecule-production information is summarized in Table 1 ▸.

### 2.2. Crystallization

His-*Gse*PNPase was crystallized using 25 mg ml^−1^ protein at a 2:1 protein:well solution ratio (drop volume 300 nl; well volume 40 µl) using the commercial MORPHEUS protein crystallization screen (Gorrec, 2009 ▸) and a Mosquito robot (SPT Labtech). Crystals were harvested and cooled in liquid nitrogen. Crystallization information is summarized in Table 2 ▸.

### 2.3. Data collection and processing

Diffraction data were collected at the Australian Synchrotron on the MX2 macromolecular crystallography beamline equipped with a Dectris EIGER 16M detector (Aragão *et al.*, 2018 ▸). From each crystal that diffracted, 720° of data were collected and processed using *X-ray Detector Software* (*XDS*; Kabsch, 2010 ▸). The reflections were merged using *AIMLESS* (Evans & Murshudov, 2013 ▸) and the most likely space group, *P*4_3_2_1_2, was determined using *POINTLESS* from the *CCP*4 suite (Winn *et al.*, 2011 ▸). An *R*_free_ set corresponding to 5% of reflections was assigned. Data-collection and processing statistics are summarized in Table 3 ▸.

### 2.4. Structure solution and refinement

A molecular-replacement search model (PDB entry 2ac7; adenosine phosphorylase from *Bacillus cereus*; A. Rinaldo-Matthis, S. Allegrini & F. Sgarrella, unpublished work), with the His-*Gse*PNPase sequence docked onto it, was used in molecular replacement to solve the structure of His-*Gse*PNPase. The His-*Gse*PNPase sequence was docked onto the model using *CHAINSAW* (Stein, 2008 ▸) and nonmatching regions were removed. Analysis of the Matthews coefficient (Matthews, 1968 ▸) revealed that there were likely to be three molecules in the asymmetric unit. Molecular replacement was completed using *Phaser* (McCoy *et al.*, 2007 ▸) in *CCP*4 and was run in expert mode to find all possible space groups within the chosen point group (*P*422), as well as to refine the solution and reduce the *R* factor. *REFMAC*5 (Murshudov *et al.*, 2011 ▸) from the *CCP*4 suite was then used to refine the solution further. The electron-density maps and model generated by the above programs were used in *Coot* (Emsley *et al.*, 2010 ▸) to manually build and refine the model. After each round of manual building, the model was refined further in *REFMAC*5 or *Phenix* (Afonine *et al.*, 2012 ▸). *R* and *R*_free_ were monitored to determine the model quality. The structure has been deposited in the PDB as entry 8d38. Refinement statistics are summarized in Table 4 ▸.

## 3. Results and discussion

### 3.1. His-*Gse*PNPase adopts a hexameric quaternary structure

**Figure 2:**
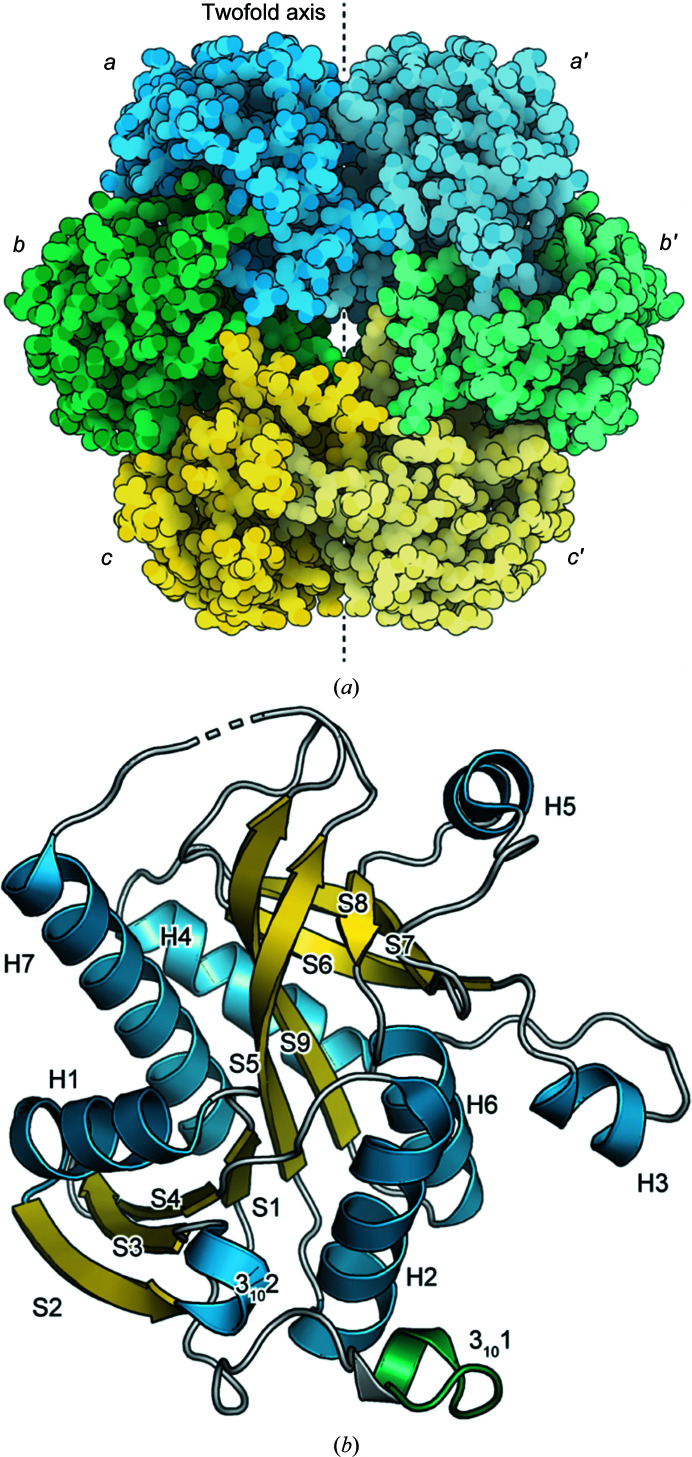
The structure of His-*Gse*PNPase. (*a*) The hexameric biological unit of PNPase. The asymmetric unit contains one-and-a-half dimers (subunits *a–c*) and the hexamer is generated by a twofold symmetry operation that produces subunits *a*′–*c*′. (*b*) The tertiary structure of a His-*Gse*PNPase subunit. Secondary-structure elements are labelled and helices are coloured blue, β-sheets yellow and loop regions white. The portion of the structure corresponding to the N-terminal tag is coloured green and features a 3_10_-helix (3_10_1).

His-*Gse*PNPase crystallized in the absence of ligands (Table 2 ▸) in space group *P*4_3_2_1_2 (Table 3 ▸) and diffracted to a resolution of 1.72 Å. Three chains (*a*–*c*) were present in the asymmetric unit, from which the hexameric biological assembly can be generated through the application of crystallographic symmetry operations (Fig. 2 ▸ *a*). The hexameric structure shows threefold symmetry through the ring-shaped hexamer and His-*Gse*PNPase can be viewed as being a trimer of dimers (*a*/*b*, *a*′/*b*′ and *c*/*c*′), like other hexameric PNPases (Narczyk *et al.*, 2021 ▸). The observed electron density for each chain is continuous, apart from residues 209–210 in chain *c* and portions of the N-terminal tag.

### 3.2. Monomeric fold

Analysis of the fold of the subunits suggests that His-*Gse*PNPase is structurally similar to other hexameric PNPases, such as the hexameric PNPase from *E. coli* (*Ec*PNPase; C^α^ r.m.s.d. of 0.614 Å; Mao *et al.*, 1997 ▸; Rinaldo-Matthis *et al.*, 2007 ▸; Grenha *et al.*, 2005 ▸). More generally, the His-*Gse*PNPase subunit structure possesses a conserved fold, with over 702 matches (C^α^ r.m.s.d. of 0–2.5 Å) in a *PDBeFold* search (Krissinel & Henrick, 2004*a* ▸,*b* ▸, 2005 ▸; Krissinel *et al.*, 2004 ▸). Its structure is most like those of *Helicobacter pylori* PNPase (Narczyk *et al.*, 2018 ▸) and the well studied *Ec*PNPase (Narczyk *et al.*, 2021 ▸), with r.m.s.d.s of 0.42–0.51 Å. The subunits of His-*Gse*PNPase are similar to each other, with r.m.s.d.s of 0.136–0.160 Å on pairwise comparison. Each subunit is comprised of seven α-helices (H1–H7) and two β-sheets (Fig. 2 ▸ *b*). The first β-sheet (S1–S4) has four strands, with S3 antiparallel, and the second sheet forms a sheet-like barrel roll (S5–S10) with S5 and S10 antiparallel. Based on analysis in SCOP, His-*Gse*PNPase is part of the phosphorylase/hydrolase-like fold superfamily, as are other PNPases (Fox *et al.*, 2014 ▸).

### 3.3. High conservation between the His-*Gse*PNPase and *Ec*PNPase active sites

**Figure 3:**
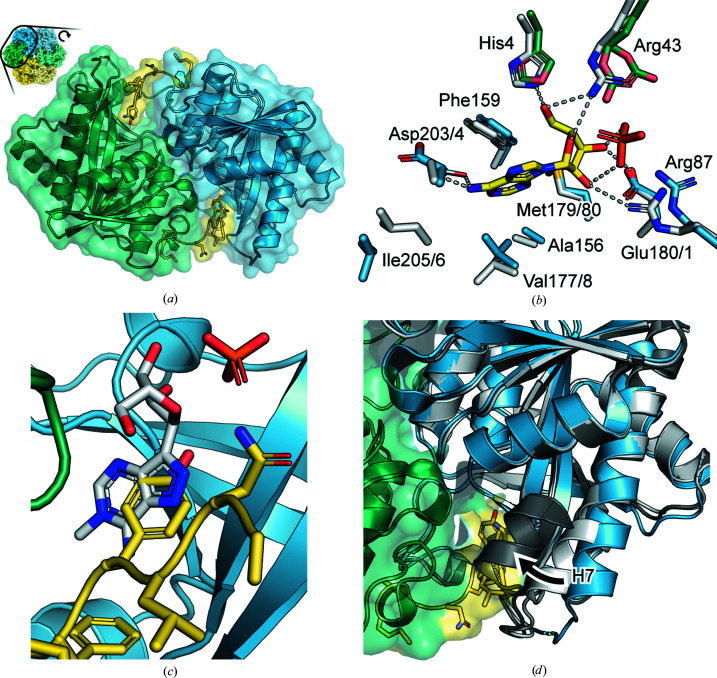
(*a*) The dimeric substructure of His-*Gse*PNPase. The two subunits are independently coloured and the regions of each corresponding to the N-terminal tag are coloured yellow. The position of the N-terminal tag indicates the approximate position of the active sites in each dimer, which sit at the interface between the subunits. (*b*) A structural overlay of *Ec*PNPase (PDB entry 1pk7, white) and His-*Gse*PNPase (subunit *a* in blue and subunit *b* in green) highlighting the conservation of the active-site residues. Adenine and phosphate from the *Ec*PNPase structure are coloured yellow. Putative hydrogen-bonding interactions between these molecules and the *Ec*PNPase residues are shown as dashed lines. (*c*) Overlay of the Tyr5 residue from the rTEV site (yellow) and the adenine base from PDB entry 1k9s (white). (*d*) The segmenting H7 in the two different positions found in *Ec*PNPase (closed, dark grey; open, white, from PDB entry 1k9s chains *A *and *D*) and the position of this region in His-*Gse*PNPase (blue; the N-terminal tag is shown in yellow).

The active sites are formed close to the interfaces between two subunits (for example subunits *a* and *b*; Fig. 3 ▸ *a*) and there are six active sites in the hexamer. The active site is mostly formed from residues from one subunit, with additional contributing residues from the other subunit in each dimer pair (Fig. 3 ▸ *b*). There is high sequence and structural conservation between the active sites of *Ec*PNPase and His-*Gse*PNPase. In *Ec*PNPase, the residues that interact with the purine base were identified as Ala156, Phe159, Val178, Met180, Asp204 and Ile206 (Bennett *et al.*, 2003 ▸), which are equivalent to Ala156, Phe159, Val177, Met179, Asp203 and Ile205, respectively, in His-*Gse*PNPase (Fig. 3 ▸ *b*). The ribose-binding site in *Ec*PNPase is primarily formed by interactions with Glu181 from one subunit and His4 from an adjacent subunit (Bennett *et al.*, 2003 ▸), which are equivalent to Glu180 and His4, respectively, in His-*Gse*PNPase (Fig. 3 ▸ *b*). The phosphate-binding site of *Ec*PNPase is comprised of two arginine residues, Arg87 from one subunit and Arg43 from an adjacent subunit, and these residues are also conserved in His-*Gse*PNPase (Fig. 3 ▸ *b*).

### 3.4. Occupation of the active site by the N-terminal tag from an adjacent subunit

One surprising aspect of the His-*Gse*PNPase structure is the presence in the active site of several residues (NLYFQ) from the nine-residue rTEV site (ENLYFQGAM) in the N-terminal tag. This portion of the rTEV site was observed to be bound in the active site of the adjacent subunit in each dimer (Fig. 3 ▸ *a*) consistently across all dimers in the hexameric structure. The interactions between the active site and the N-terminal tag are dominated by the Tyr residue in the rTEV recognition site. This residue is oriented in a similar way to the purine-base part of the substrate (Fig. 3 ▸ *c*). This similarity in binding pose suggests that the hydrophobic stacking interactions that this residue makes with active-site residues may contribute to holding the N-terminal tag in its position bound to the active site and thus also potentially prevent access to and cleavage by the protease which cuts between Gln and Gly in the sequence.

### 3.53. N-terminal tag occupation prevents the His-*Gse*PNPase active site from closing

PNPase structures feature an α-helix (H7, using the α-helix numbering for *Ec*PNPase; Mao *et al.*, 1997 ▸) that can segment into two parts to close over the active site (Štefanić *et al.*, 2017 ▸). In His-*Gse*PNPase H7 consists of residues Val200–Gln217. In *Ec*PNPase two different active-site conformations have been observed, denoted as ‘open’ (nonsegmented form) and ‘closed’ (segmented form) (Štefanić *et al.*, 2018 ▸). In the closed conformation, this loop abrogates access to the active site (Koellner *et al.*, 2002 ▸). In the structure of His-*Gse*PNPase, the conformation of this loop in all three subunits is most consistent with the *Ec*PNPase ‘open’ conformation (Fig. 3 ▸ *d*), with the occupation of the active site by the tag precluding the ability of the helix to segment and adopt the ‘closed’ conformation.

### 3.6. Interactions of the N-terminal tag with the active site may affect enzyme function

Before considering the functional implications of N-terminal tag binding in the active site, it is first necessary to investigate whether this is simply a crystallization artefact driven by proximity effects and entropic factors. We therefore attempted to cleave the tag and purify the untagged form, with a view to performing joint structural and functional characterization of the tagged and untagged forms. However, regardless of the experimental conditions, only mixed populations of cleaved and uncleaved subunits were obtained, which were predominantly uncleaved. This result suggests that the tag may be difficult to remove because His-*Gse*PNPase exists in a tag-bound form in solution, precluding access of the protease to the cleavage site.

The strong affinity of the tag for the active site in the crystallized form is supported by the results of crystallization screening experiments in the presence of high concentrations of the PNPase inhibitor acyclovir and the substrate 7-methylguanine, which show that neither substrate is capable of displacing the tag from the active site. However, UV–Vis activity assays tell a different story, demonstrating that N-terminally tagged His-*Gse*PNPase is catalytically active in solution (data not shown). The enzymatic activity implies that the active site must be at least somewhat accessible to the substrate under standard assay conditions. However, it is important to keep in mind that while substrates can compete with the tag for active-site binding in solution, the reverse may also be true, *i.e.* the tag may compete with substrates, potentially affecting the activity of the enzyme.

Taken together, this evidence suggests that it is most likely that His-*Gse*PNPase exists in a tag-bound form in both the solution and crystalline phases, but with a lower affinity in solution due to entropic favourability of the disordered unbound form partially counterbalancing the binding enthalpy. This situation, along with the more dynamic nature of proteins in solution in general, may explain why substrate binding is observed in activity assays but not in crystallization screening.

### 3.7. Comparison with related superfamilies reveals a fold that can accommodate peptide binding

Although proximity effects undoubtedly contribute to promoting tag binding, *Gse*PNPase may also have a native affinity for peptide substrates and an ability to accommodate peptide binding. Analysis in SCOP (Andreeva *et al.*, 2014 ▸, 2020 ▸) of the purine/uridine phosphorylase superfamily that has the same phosphorylase/hydrolase-like fold as the PNPases reveals members that cleave peptide substrates (for example zinc-dependent exopeptidases, pyrrolidone carboxyl peptidase and the peptidyl-tRNA hydrolase-like and HybD-like superfamilies). Structural alignments between His-*Gse*PNPase and superfamily members, including carboxy­peptidase T in complex with a protein inhibitor (PDB entry 1dtd; Reverter *et al.*, 2000 ▸), suggest that the location of the active site is conserved and the fold generally accommodates peptide binding. Notably, peptides generated from snake venom have been shown to be effective inhibitors of PNPase from *Plasmodium falciparum* (Martins *et al.*, 2019 ▸), suggesting that not only can PNPases bind peptides by that they may also be a potential route for the design of new PNPase inhibitors.

## Supplementary Material

PDB reference: purine nucleoside phosphorylase, 8d38

## Figures and Tables

**Table 1:** Macromolecule-production information

Source organism	*Geobacillus stearothermophilus*
DNA source	Synthetic DNA
Cloning vector	pET-30a
Expression vector	pET-30a
Expression host	*Escherichia coli* BL21 (DE3)
Complete amino-acid sequence of the construct produced	MHHHHHHSSGLVPRGSGMKETAAAKFERQHMDSPDLGTGSENLYFQGAMMSVHIGAKAGEIAERILLPGDPLRAKYIAETFLEGAVCYNEVRGMLGFTGTYKGERISVQGTGMGVPSISIYVNELIQSYGVKTLIRVGTCGAIQPDVRVRDVILAMSASTDSNMNRLIFRGRDYAPTADFHLLRTAYEVGVEKGLALKVGNVFTADMFYNDEPNWETWARYGVLAVEMETAALYTLAAKFGCRALSVLTVSDHILTGEETTAEERQMTFNEMIEVALEAAIRNGA

**Table 2:** Crystallization

Method	Vapour diffusion
Plate type	Sitting drop
Temperature (K)	291
Protein concentration (mg ml^−1^)	25
Buffer composition of protein solution	50 m*M* HEPES pH 7.6, 100 m*M* NaCl
Composition of reservoir solution	0.06 *M* magnesium chloride hexahydrate, 0.06 *M* calcium chloride dihydrate, 0.1 *M* Tris (base), 30%(*v*/*v*) Bicine pH 8.5, 40%(*v*/*v*) ethylene glycol, 20%(*w*/*v*) PEG 8000
Volume and ratio of drop	300 nl; 2:1 protein:reservoir solution
Volume of reservoir (µl)	40

**Table 3:** Data collection and processing Values in parentheses are for the outer shell.

Diffraction source	MX2 beamline, Australian Synchrotron
Wavelength (Å)	0.95374
Detector	Dectris EIGER 16M
Total rotation range (°)	720
Space group	*P*4_3_2_1_2
*a*, *b*, *c* (Å)	102.96, 102.96, 167.76
α, β, γ (°)	90, 90, 90
Resolution range (Å)	49.220–1.720 (1.750–1.720)
Total No. of reflections	5180774 (256581)
No. of unique reflections	96171 (4713)
Completeness (%)	100.000 (99.900)
Multiplicity	53.900 (54.400)
Mn(*I*) half-set correlation (CC_1/2_)	1.00 (0.599)
〈*I*/σ(*I*)〉	18.500 (0.5)†
*R*_r.i.m._	0.157 (9.227)
*R*_p.i.m._	0.021
Overall *B* factor from Wilson plot (Å^2^)	34.170

† The mean *I*/σ(*I*) in the outer shell falls below 2.0 at resolutions above 1.92 Å. Analyses of merged CC_1/2_ correlations between intensity estimates from half data sets (Karplus & Diederichs, 2015 ▸) were used to influence the high-resolution cutoff for data processing.

**Table 4:** Structure refinement

Resolution range (Å)	46.0500–1.7200 (1.7400–1.7200)
Completeness (%)	99.5
σ Cutoff	*F* > 1.330σ(*F*)
No. of reflections, working set	90727 (2976)
No. of reflections, test set	4907 (149)
Final *R*_cryst_	0.196 (0.4449)
Final *R*_free_	0.224 (0.4695)
No. of non-H atoms	
Total	5755
Protein	5487
Ions	2
Solvent	266
R.m.s. deviations	
Bond lengths (Å)	0.013
Angles (°)	1.177
Average *B* factors (Å^2^)	
Protein	42.58
Ions	34.29
Solvent	43.23
Ramachandran plot	
Most favoured (%)	97.45
Allowed (%)	2.55
